# Bridging the analog divide: a comparison of printed X-ray films and digital images when using computer-aided detection software for tuberculosis screening

**DOI:** 10.1186/s44263-025-00237-8

**Published:** 2026-01-13

**Authors:** Andrew J. Codlin, Thang P. Dao, Binh H. Nguyen, Luan N. Q. Vo, Rachel J. Forse, Ha T. M. Dang, Lan H. Nguyen, Hoa B. Nguyen, Luong V. Dinh, Kristi Sidney Annerstedt, Johan Lundin, Knut Lönnroth

**Affiliations:** 1 Friends for International TB Relief, Ha Noi, Viet Nam; 2 Department of Global Public Health, Karolinska Institutet, Stockholm, Sweden; 3 Pham Ngoc Thach Hospital, Ho Chi Minh City, Viet Nam; 4 National Lung Hospital, Ha Noi, Viet Nam; 5 Institute for Molecular Medicine Finland (FIMM), University of Helsinki, Helsinki, Finland

**Keywords:** Computerized image analysis, Computer-aided detection (CAD), Chest radiography, Tuberculosis (TB)

## Abstract

**Background:**

Computer-aided detection (CAD) software provides scalable, standardized chest X-ray (CXR) interpretation, helping address the global shortage of radiologists and inter-reader variability. Printed X-ray films remain common in many low-resource settings, yet most CAD software can only process Digital Imaging and Communications in Medicine (DICOM) files. Genki software (DeepTek, India) is one of the few World Health Organization (WHO)–recommended CAD software capable of interpreting both DICOM files and photographs of printed X-ray films (Joint Photographic Experts Group [JPEG] files), but its performance using JPEG files has not been independently evaluated.

**Methods:**

We evaluated Genki software using a test library of 1466 CXR images from adults screened for tuberculosis (TB) in Ho Chi Minh City, Viet Nam. Each participant’s TB status was determined using a composite reference standard, based on radiological findings and Xpert MTB/RIF Ultra testing. Each CXR image was blindly re-read by 10 human readers and processed by Genki software using both DICOM and JPEG files. Genki software performance was evaluated using median abnormality scores, area under the receiver operating characteristic curves (AUC), and sensitivity/specificity comparisons at different abnormality score thresholds.

**Results:**

Genki software abnormality scores were significantly higher when using JPEG files, but this did not translate into significant differences in AUCs between the file types (DICOM AUC = 0.94 vs JPEG AUC = 0.92, *p* = 0.190). When abnormality score thresholds were calibrated to match average human reader sensitivity (79.0%), Genki achieved significantly higher specificity with both DICOM (95.2% vs 84.8%, *p* < 0.001) and JPEG (92.1% vs 84.8%, *p* < 0.001) files. When the software’s abnormality score thresholds were calibrated to achieve 90% sensitivity, Genki maintained high specificity with both DICOM (89.3%) and JPEG (81.1%) file types, meeting the minimum Target Product Profile (TPP) criteria for a high-sensitivity, high-specificity screening test.

**Conclusions:**

Genki software performs comparably when interpreting DICOM and JPEG files, outperforming human readers and meeting TPP criteria with both file types. This capability enhances its usability in resource-limited settings where digital infrastructure is lacking, supporting its broader deployment for TB screening. Further research is needed to assess real-world implementation feasibility and performance in diverse populations and clinical environments.

**Supplementary Information:**

The online version contains supplementary material available at 10.1186/s44263-025-00237-8.

## Background

Tuberculosis (TB) remains the leading cause of death from a single infectious agent globally, with an estimated 10.7 million people falling ill and 1.23 million dying from TB in 2024 [1]. A key driver of this continued burden is the low global treatment coverage rate, which is estimated to have been just 78% in 2024. One reason TB detection can be delayed or missed is the high proportion of people with asymptomatic or subclinical TB; over 80% of people with TB do not report a cough lasting more than 2 weeks (the most common TB referral criteria [2]), and over 60% report no cough at all [3]. The World Health Organization (WHO) now recommends the use of symptom-independent screening tools [4], such as chest X-ray (CXR), which can be highly sensitive for detecting pulmonary abnormalities and may be used for TB screening in both community and clinical settings.

However, the feasibility and efficiency of CXR screening for TB has been limited by two critical prerequisites: the availability of radiography equipment and access to qualified human resources for CXR image interpretation. The availability of radiography equipment in high TB burden, low- and middle-income countries (LMICs) is highly variable, with many facilities still reliant on older analog machines. This ‘analog divide’ poses challenges for large-scale screening; analog systems require films, chemicals, and darkroom infrastructure, all of which increase costs and slow down workflows. The transition from analog to digital radiography is hindered by high capital and maintenance costs, unreliable electricity supply, limited local technical support, and uneven policy and regulatory frameworks, though adoption is underway in most high TB burden, LMICs [5]. CXR screening depends heavily on human readers who may not always be available [6] or consistently accurate [7]. Interpretation quality can vary significantly based on reader experience, training, and workload. In many settings, trained radiologists are scarce [6, 8], and reporting radiographers or non-specialist clinicians are often tasked with interpreting CXRs, contributing to missed opportunities for TB diagnostic testing.

Computer-aided detection (CAD) software, which offers standardized, scalable, and rapid assessments of CXR images, has emerged as a promising technology to address these challenges. In 2021, the WHO issued guidelines recommending the use of three CAD software (e.g., CAD4TB [Delft Imaging, The Netherlands], INSIGHT CXR [Lunit, South Korea], and qXR [Qure.ai, India]) as a replacement for human readers during TB screening [4]. Since then, Genki CAD software (DeepTek, India) has been independently assessed in multiple comparative evaluations [9–12] and in standalone studies [13, 14], and its performance has been shown to meet the original Target Product Profile (TPP) criteria for a community-based TB referral test (e.g., ≥ 90% sensitivity and ≥ 70% specificity) [15]. In 2025, the WHO updated its guidelines to recommend three additional CAD software solutions: DrAid (VinBrain, Viet Nam), Genki, and InferRead DR Chest (InferVision, Germany) [16].

Most CAD software can only process Digital Imaging and Communications in Medicine (DICOM) files, which limits their usability in many high TB burden, LMICs. Even in facilities equipped with computed radiography (CR) or digital radiography (DR) systems, printed X-ray films are often still shared with patients or they are used for record keeping when digital storage and transfer infrastructure is lacking. In such contexts, CAD software capable of interpreting photographs of printed X-ray films or Joint Photographic Experts Group (JPEG) files would offer a practical solution to the lack of digital radiology services. Genki is one of the few WHO-recommended CAD software with this functionality, but it has never been independently evaluated.

Therefore, we conducted an assessment of the Genki software to compare its performance interpreting CXR images for TB-related abnormalities using both DICOM and JPEG files.

## Methods

### Mobile CXR screening

A total of 133 mobile CXR screening events were hosted across five districts of Ho Chi Minh City, Viet Nam between July 2022 and March 2023. The standardized methods for participant mobilization and evaluation have been described elsewhere [17]. At the events, participants completed a questionnaire about their TB symptoms and risk factors, and were then screened by CXR. CXR images were interpreted on the spot using two independent methods. An on-site radiologist provided an interpretation and the CXR images were processed with qXR CAD software (Version 3) in parallel; the on-site radiologists were blinded to the qXR CAD software outputs, resulting in two independent, but paired CXR interpretations. Participants who had any abnormal result—either from the radiologist and/or the qXR CAD software (abnormality score ≥ 0.50), even when the results were discordant—were indicated for sputum testing using the Xpert MTB/RIF Ultra (Xpert-Ultra) assay. Participants diagnosed with drug-sensitive TB were linked to treatment at the nearest District TB Unit, and those diagnosed with drug-resistant TB were linked to treatment at the Pham Ngoc Thach TB Reference Hospital.

Two types of mobile CXR screening events were conducted, each designed to meet distinct objectives of the project which funded the screening activities (e.g., maximizing the yield of TB or the scale/coverage of screening activities). Thirty-five (26.3%) screening events aimed at maximizing the yield of TB were organized using an ultraportable DR system (Xair, Fujifilm, Japan) [18]. At this type of screening event, the target population comprised household contacts, people with HIV, and TB program and laboratory staff, and an average of 45 participants were screened with CXR per event. Ninety-eight (73.7%) screening events aimed at maximizing the scale/coverage of screening were organized using a DR system (DRTECH, South Korea) mounted inside a truck. At these types of screening events, the target population comprised primarily elderly and symptomatic individuals, and an average of 260 participants were screened with CXR per event. As a result, the average yield of TB at this second type of screening event was significantly lower than at the first, more targeted type of screening event.

At the end of each mobile CXR screening event, DICOM files were exported from the radiography system to a study hard drive for storage.

### Test library creation

**Fig. 1:**
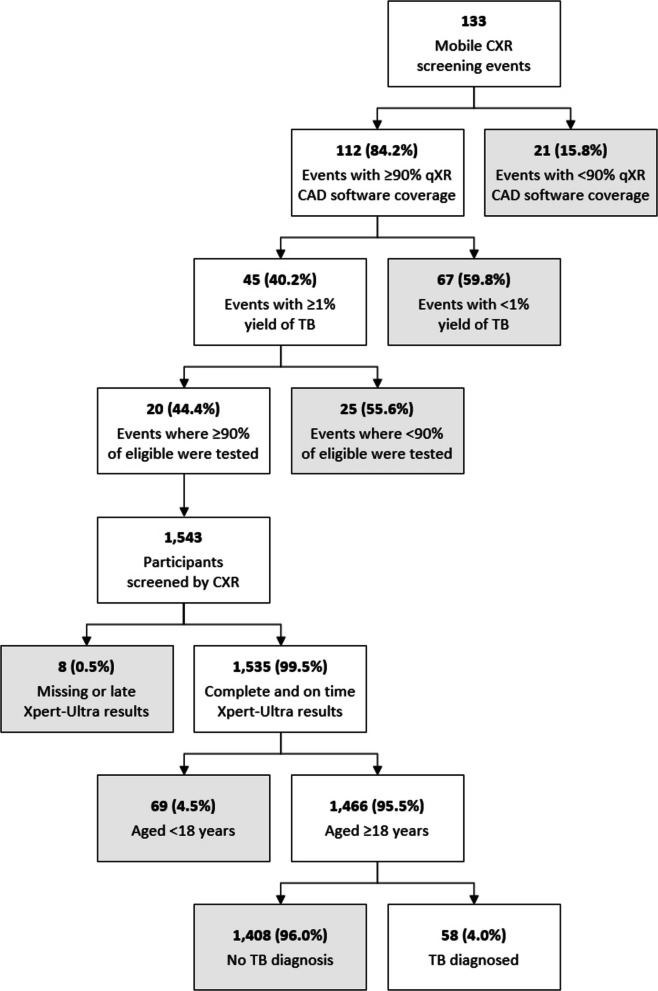
Flow diagram showing the test library’s creation. Both event-level and participant-level inclusion criteria were applied to curate the test library. ACF events were included only if they had high qXR coverage, a TB yield ≥ 1%, and ≥ 90% Xpert-Ultra testing among eligible participants. Participant-level criteria were then applied to the 1543 individuals screened at the 20 eligible ACF events, excluding those with missing or delayed Xpert-Ultra results and those aged < 18 years

A DICOM file test library was constructed using data from the aforementioned mobile CXR screening events, ensuring that the library’s participant demographics reflected those of the individuals screened in the community. Mobile CXR screening data were first filtered by event-level inclusion criteria (Fig. 1) to include only events with high qXR coverage, high yield of TB, and high testing coverage among eligible participants. These criteria reduced the overall size of the test library, making it feasible to have TB clinicians re-read the CXR images for this evaluation, and ensured that a composite TB reference standard could be consistently applied to all participants. From a total of 133 mobile CXR screening events, 112 (84.2%) achieved a real-time qXR software coverage of ≥ 90%, 45 (40.2%) achieved a yield of TB ≥ 1%, and 20 (44.4%) achieved an Xpert-Ultra testing rate of ≥ 90% among eligible participants.

A total of 1543 participants were screened with CXR at these 20 events. A series of individual-level inclusion criteria were then applied to finalize the test library’s participants. Eight (0.5%) individuals were excluded because they were missing Xpert-Ultra results or were tested > 30 days after their CXR screen, meaning it would be impossible to construct a TB reference standard for these individuals. In addition, 69 (4.5%) individuals were excluded because they were aged less than 18 years. The final testing library included 1,466 CXR images of adult participants.

A mix of radiologists, qXR CAD software, and Xpert-Ultra test results were used to establish a composite reference standard for all test library participants (Supplementary material 1: Fig. S1). Individuals with a positive Xpert-Ultra result after any abnormal CXR result (either from the radiologist and/or the qXR CAD software) were classified as having TB. No participants were tested on Xpert-Ultra after a normal CXR result. Those with either two normal CXR results (from both the radiologist and the qXR CAD software) or a negative Xpert-Ultra result after any abnormal CXR result were classified as not having TB.

All of the test library’s DICOM files were de-identified by project staff using a custom-built Python tool which replaced their metadata with study identifiers. Individual-level demographic, symptom, and Xpert-Ultra test data were then extracted from the electronic data system used during the mobile CXR screening events [19].

### Test library printing and photographing

The test library’s de-identified CXR images were printed onto 20 × 25 cm X-ray films using a single printer with a 14-bit depth (Carestream TRIMAX TX55 Laser Imager, United States). The X-ray films were then photographed using the 48-megapixel rear camera of a Galaxy A12 mobile phone with a 24-bit depth (Samsung, South Korea). The X-ray film photographs were taken in a standardized fashion in a darkened room, with the X-ray film mounted on a light box and the mobile phone stabilized by a tripod at a distance of roughly 0.5 m. With this setup, the X-ray film photographs contain only the film itself, without including the exposed light box or any unwanted light.

### Test library reading

The test library’s de-identified DICOM files were sent to 10 TB clinicians working at public (*n* = 5) and private (*n* = 5) health facilities across Ho Chi Minh City for blinded reading. These individuals had varied specializations, but all were responsible for CXR interpretation and TB care within their respective health facilities (Supplementary material 1: Table S1); 50% of the readers had obtained a certificate in radiology between 0 and 14 years before the CXR reading for this evaluation. They interpreted the test library’s CXR images using a four-category reporting system: ‘TB-related abnormality in the lungs’, ‘Other abnormality in the lungs’, ‘Abnormality outside the lungs’, and ‘Normal’.

The test library’s de-identified DICOM files and photographs of the printed X-ray films (JPEG files) were securely transferred to DeepTek and processed with Genki software (Version 3.4.2). DeepTek then returned paired continuous abnormality scores for analysis.

### Statistical analyses

Descriptive statistics of the test library were calculated and differences in TB status were compared using the chi-square test. The median Genki CAD software abnormality scores were calculated by file type and TB status and were compared using the Mann–Whitney *U* test. The receiver operating characteristic (ROC) curves were plotted for each file type, and the area under the curves (AUC) was calculated and compared, using the composite TB status as the reference standard [20]. The average sensitivity and specificity of the 10 human readers were calculated in two ways: with only ‘TB-related abnormality in the lungs’ being considered as abnormal, and then with any abnormalities in or outside the lungs being considered as abnormal. A Genki CAD software abnormality score threshold that corresponds to the average sensitivity of the human readers was then selected for the two different human interpretations and for each file type, and software specificity was calculated at the different thresholds. Then, a Genki software abnormality score threshold corresponding to 90% sensitivity was selected for each file type and software specificity was calculated at the different thresholds, to assess the software’s performance against the updated TPP criteria for a high-sensitivity and high-specificity TB screening test (e.g., ≥ 90% sensitivity and ≥ 80% specificity) [21]. Sensitivities and specificities at the aforementioned thresholds were compared using generalized estimating equations [22].

## Results

### Test library characteristics

Of the 1,466 participants in the test library, 58 (4.0%) had TB according to the evaluation’s composite reference standard definition, as described in the methods section (Table 1). The test library’s participants were 56.3% male, and males had TB at a significantly higher rate than females (5.3% vs 2.2%, *p* < 0.001). The median age of test library participants was 49 years; younger participants aged 18–54 years constitute almost two thirds of the test library; however, there are no significant differences in the rate of TB by age. Just 9.6% of the test library’s participants reported not having social health insurance (SHI). Those without SHI were significantly more likely to have TB than those with SHI (7.9% vs 3.5%, *p* = 0.014). 19.3% of test library participants reported having TB symptoms, and these individuals had a significantly higher rate of TB than those who were asymptomatic (14.5% vs 1.4%, *p *< 0.001). One third of the test library’s participants reported having contact with someone who had TB, and they had significantly higher rates of TB than non-contacts (8.6% vs 1.7%, *p* < 0.001). 17.1% of the participants had a past history of TB, and this group had a significantly higher rate of TB (10.8% vs 2.6%, *p* < 0.001). A small proportion (4.8%) of the test library’s participants report having diabetes, but there were no significant differences in rates of TB between persons with diabetes and non-diabetics. One quarter of the test library’s participants reported having HIV, and this cohort had a significantly lower rate of TB than those who did not have HIV (2.2% vs 4.5%, *p* = 0.047). 57.5% of the test library’s CXR images were captured using an ultraportable Xair device, and participants screened with this radiography system had a significantly higher rate of TB (5.9% vs 1.3%, *p* < 0.001).

**Table 1:** Description of the test library’s participants

	All participants	TB	No TB	*p*-value
All participants	1,466	58 (4.0%)	1408 (96.0%)	N/A
Demographic factors				
Sex				
Male	825 (56.3%)	44 (5.3%)	781 (94.7%)	0.002
Female	641 (43.7%)	14 (2.2%)	627 (97.8%)	
Age, median (interquartile range)	49 (38–61)	49 (39–58)	49 (38–61.5)	0.875
18–54 years	884 (60.3%)	36 (4.1%)	848 (95.9%)	0.779
≥ 55 years	582 (39.7%)	22 (3.8%)	560 (96.2%)	
Lacking health insurance	141 (9.6%)	11 (7.8%)	130 (92.2%)	0.014
Presence of TB symptoms				
Cough (C)	251 (17.1%)	37 (14.7%)	214 (85.3%)	< 0.001
Fever (F)	39 (2.7%)	14 (35.9%)	25 (64.1%)	< 0.001
Weight loss (WL)	58 (4.0%)	15 (25.9%)	43 (74.1%)	< 0.001
Night sweats (NS)	20 (1.4%)	6 (30.0%)	14 (70.0%)	< 0.001
C, F, WL, and/or NS	283 (19.3%)	41 (14.5%)	242 (85.5%)	< 0.001
TB risk factors				
Contact of person with TB	475 (32.4%)	41 (8.6%)	434 (91.4%)	< 0.001
Past history of TB	251 (17.1%)	27 (10.8%)	224 (89.2%)	< 0.001
Diabetes	71 (4.8%)	3 (4.2%)	68 (95.8%)	0.905
HIV	364 (24.8%)	8 (2.2%)	356 (97.8%)	0.047
Radiography system				
Ultraportable (Xair)	843 (57.5%)	50 (5.9%)	793 (94.1%)	< 0.001
X-ray truck (DRTECH)	623 (42.5%)	8 (1.3%)	615 (98.7%)	

### Genki software performance

Table 2 shows the median Genki software abnormality scores by file type and TB status. For all participants, the median abnormality score is significantly higher for the photographs of the printed X-ray films (JPEG files 0.01 [0.01–0.17] vs DICOM files 0.01 [0.00–0.06], *p* < 0.001). There is no significant difference in median abnormality scores between the file types for participants with TB (JPEG files 0.75 [0.47–0.85] vs DICOM files 0.78 [0.47–0.90], *p* = 0.951), but there is a significant difference among those who did not have TB (JPEG files 0.03 [0.01–0.15] vs DICOM files 0.01 [0.00–0.05], *p* < 0.001).

**Table 2:** Comparison of median Genki abnormality scores, by file type and TB status

	All participants		TB		No TB	
File type	Median score (IQR)	*p*-value	Median score (IQR)	*p*-value	Median score (IQR)	*p*-value
DICOM files	0.01 (0.00–0.06)	< 0.001	0.78 (0.47–0.90)	0.951	0.01 (0.00–0.05)	< 0.001
JPEG files	0.01 (0.01–0.17)		0.75 (0.47–0.85)		0.03 (0.01–0.15)	

**Fig. 2:**
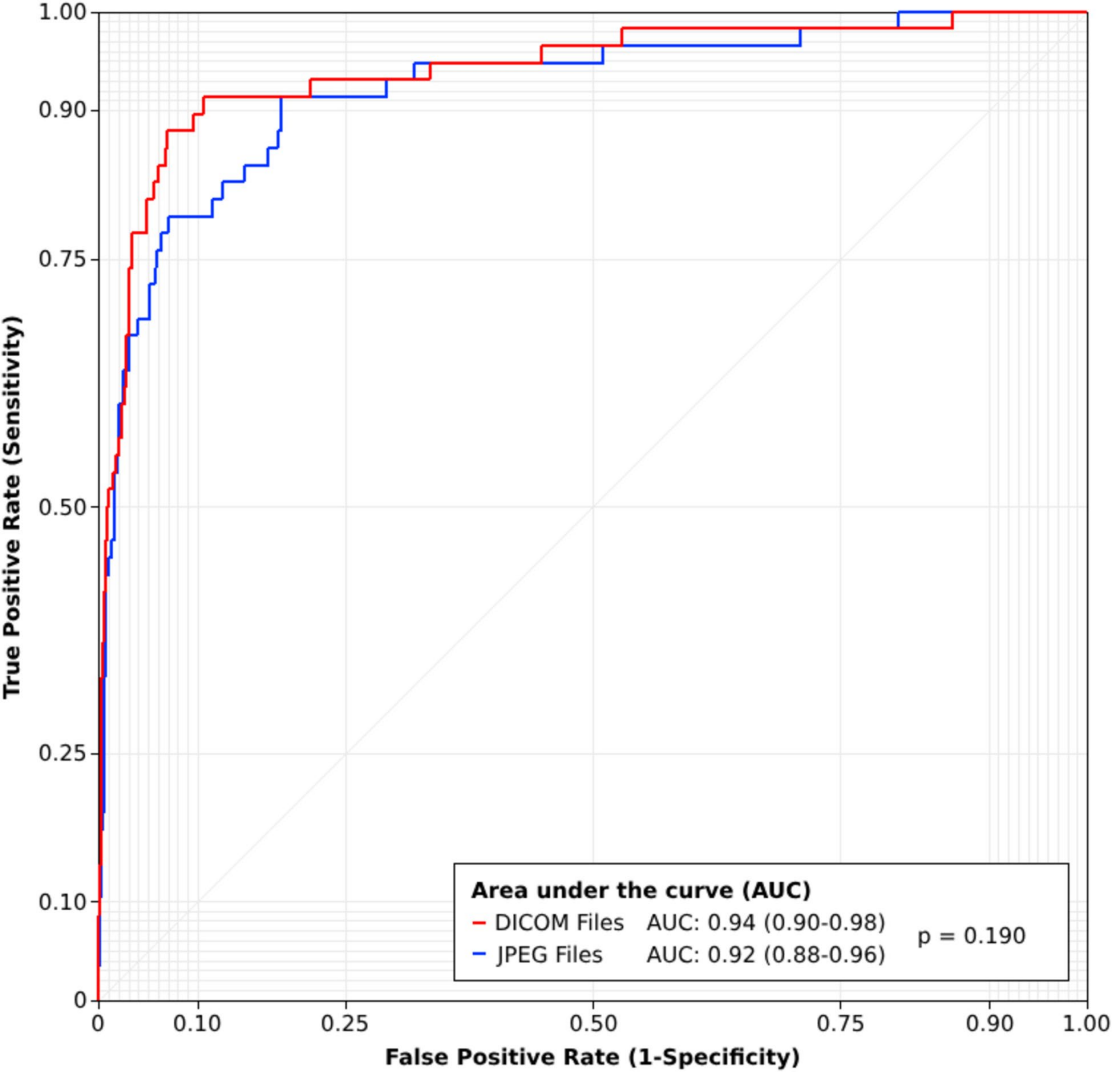
Plot of receiver operating characteristic (ROC) curves and area under the curves (AUC), by file type. The red curve represents DICOM file performance, while the blue curve represents JPEG file performance. The callout box presents the AUC values for each file type and a *p*-value comparing the two curves

Figure 2 shows the plot of the ROC curves for each file type. There is no significant difference in the AUC for the Genki CAD software when processing either DICOM or JPEG files (0.94 vs 0.92, *p* = 0.190).

The 10 human readers achieved an average sensitivity of 79.0% and a specificity of 84.8% when considering only their ‘TB-related abnormality in the lung’ classifications as abnormal (Table 3); this criterion is the standard of care CXR interpretation for indicating follow-on diagnostic testing in Viet Nam [23]. At thresholds of 0.406 and 0.410, the Genki software achieves a similar sensitivity as the human readers with the DICOM (79.3%, *p* = 0.922) and JPEG files (79.3%, *p* = 0.938) respectively. The corresponding specificity for the Genki software is significantly higher than the human readers, whether using the DICOM (84.8% vs 95.2%, *p* < 0.001) or JPEG files (84.8% vs 92.1%, *p *< 0.001). However, the software’s specificity with JPEG files is significantly lower than with DICOM files (95.2% vs 92.1%, *p* < 0.001).

**Table 3:** Genki software performance matching the average human reader sensitivity and at approximately 90% sensitivity, by file type

Reader	Score	Sensitivity	*p*-value		Specificity	*p*-value	
Human readers/abnormal TB	N/A	79.0% (75.4–82.2)	*Ref*	N/A	84.8% (84.2–85.3)	*Ref*	N/A
Genki/DICOM files	0.406	79.3% (66.7–88.8)	0.922	*Ref*	95.2% (93.9–96.2)	< 0.001	*Ref*
Genki/JPEG files	0.410	79.3% (66.7–88.8)	0.938	1.000	92.1% (90.6–93.5)	< 0.001	< 0.001
Human readers/any abnormal	N/A	87.8% (84.8–90.3)	*Ref*	N/A	66.3% (65.6–67.1)	*Ref*	N/A
Genki/DICOM files	0.250	87.9% (76.7–95.0)	0.958	*Ref*	90.9% (89.3–92.4)	< 0.001	*Ref*
Genki/JPEG files	0.240	87.9% (76.7–95.0)	0.962	1.000	81.6% (79.5–83.6)	< 0.001	< 0.001
Configured for updated TPP							
Genki/DICOM files	0.200	91.4% (81.0–97.1)	*Ref*		89.3% (87.5–90.8)	*Ref*	
Genki/JPEG files	0.230	91.4% (81.0–97.1)	1.000		81.1% (79.0–83.1)	< 0.001	

When considering any abnormality in or outside the lungs, the 10 human readers achieved an average sensitivity of 87.8% and a specificity of 66.3%. At thresholds of 0.250 and 0.240, the Genki software achieved a similar sensitivity to the human readers with the DICOM (*p* = 0.958) and JPEG files (*p* = 0.962), respectively. The corresponding specificity for the Genki software remains significantly higher than the human readers, whether using the DICOM (66.3% vs 90.9%, *p *< 0.001) or JPEG files (66.3% vs 81.6%, *p* < 0.001). Again, the software’s specificity with JPEG files is significantly lower than with DICOM files (90.9% vs 81.6%,* p* < 0.001).

When the threshold is further lowered to 0.200 for DICOM files, the software achieves a sensitivity of 91.4% and a specificity of 89.3%, exceeding the updated TPP criteria for a high-sensitivity and high-specificity screening test. At a threshold of 0.230 for the JPEG files, the software achieves a sensitivity of 91.4% and a specificity of 81.5%, also achieving the updated TPP criteria, despite specificity being significantly lower than with DICOM files (*p* < 0.001).

## Discussion

Genki software demonstrated comparable performance when processing either DICOM files or photographs of printed X-ray films (JPEG files). There were no significant differences in the AUCs between file types, and the software outperformed human readers who were responsible for TB care in the evaluation’s setting. Furthermore, the Genki software could be calibrated to achieve the minimum TPP criteria for a high-sensitivity and high-specificity TB screening tool when processing either DICOM and JPEG files.

The findings of this evaluation support the WHO’s recent CAD guideline revision [16], which added Genki to its list of recommended CAD software for use during TB screening. While the current evidence supports deploying Genki software as a replacement for human readers, the feasibility and added value of a software deployment will only be understood through prospective, head-to-head comparisons with human readers in a routine setting. A CAD software deployment is likely to have the largest added value when replacing reporting radiologists and non-specialist clinicians for reading CXR images, as these groups are likely to have lower accuracy than certified radiologists [24]. This evaluation highlights that human readers with varying specialties and experience levels, but who have been tasked with CXR interpretation in their health facilities, missed 21% of people with TB, underscoring the potential of CAD software to improve detection TB rates in routine care settings. Beyond this, there is a need to pilot and evaluate innovative CAD software deployment strategies, such as CAD parallel models focused on maximizing screening sensitivity in conjunction with human readers [25], CAD first/triage models focused on optimizing reader workloads [26], and CAD-guided specimen pooling to reduce follow-on diagnostic testing [27]. These approaches could offer significant cost and workflow advantages, and may represent more practical and strategically valuable applications of CAD software than simply using it as a replacement for human readers.

Over time, CR and DR systems will become more commonplace, owing to the resulting productivity gains and their ever declining costs [28], and this will naturally facilitate the scale-up of CAD software during TB screening. However, during the transition phase, the ability to process photographs of printed X-ray films can further facilitate the scale-up of this technology. In low-resource and low-volume sites, there may be limited ability or incentive to transition to a CR or DR system, resulting in continued reliance on printed X-ray films. In many high TB burden settings, X-ray films are used for record keeping and/or transport between sites, even when a CR or DR system is present, as they lack adequate digital infrastructure for secure image storage and interconnectedness within the health system, particularly in the private sector. Many people paying for private radiology services will take a printed X-ray film to their chosen clinician for interpretation and follow-on care prescription. In such contexts, the Genki software’s ability to process JPEG files could lead to improved CXR interpretation quality and TB screening coverage. However, the scalability of this technology will depend heavily on the deployment model and cost of the Genki CAD software, as the sites most likely to benefit from JPEG processing often have limited infrastructure and may be highly price sensitive. At the time of publishing, Genki CAD software was not included in the Global Drug Facility’s product catalogue [29] or the Global Fund’s Wambo procurement platform [30], meaning prices were not publicly available.

Although the Genki CAD software met the TPP criteria when using photographs of printed X-ray films, its specificity was significantly lower compared to processing DICOM files. Thus, the processing of JPEG files to prospectively indicate follow-on diagnostic testing could result in suboptimal utilization of finite laboratory capacity. However, this risk could be mitigated through threshold optimization, for which there are several methods now recommended [31, 32]. But it is important to note that the Genki software’s specificity when using JPEG files was significantly higher than that of the human readers it would replace or support, meaning that, in practice, its use could actually result in fewer follow-on diagnostic tests. The CAD software literature often overlooks how these tools would be used in practice, and instead emphasizes benchmarking performance against high standards which are often not equally applied to human readers. This highlights the importance of contextualizing CAD software performance within real-world implementation settings and use cases, where even less-than-ideal improvements over existing practices can yield substantial public health benefits.

CXR film photographs were captured in a highly standardized fashion during this evaluation, in a darkened room, with films mounted on a light box and using a high-quality camera stabilized on a tripod. In real-world clinical settings, such standardization may be more difficult to achieve. It is currently unclear how ambient lighting, poor alignment and distancing between the camera and X-ray film (e.g., tilting left-to-right and/or up-to-down), lower resolution photographs and the bleeding of text over the lung field on a printed film will affect the performance of the Genki software. Now that proof of concept has been established under standardized conditions, the software’s performance using JPEG files should be further evaluated prospectively, in real-world settings to understand how the aforementioned factors affect its performance, as well as establishing operational feasibility. Rotation and misalignment of a participant can affect the quality of CAD software interpretation even when processing DICOM files. Therefore, CAD software developers may consider developing built-in image quality and subject positioning checks which prompt the user to recapture a CXR image or photograph when poor image quality is detected.

CXR interpretation for TB screening in children remains a challenge and thus this key population is subject to a higher risk of having their CXRs declared as a false normal than adults [33]. All participants in this evaluation’s test library were adults, and WHO’s recent CAD guidelines revision only recommends CAD software use in individuals aged 15 and over [16]. Genki software performance has not been independently assessed in children, and only a small number of independent evaluations have been conducted among children for other WHO-recommended CAD software, which have shown only modest or sub-optimal performance [34–36]. This highlights the need for CAD software developers to build and train models specifically using pediatric CXR data. Then age-specific validation studies are needed to ensure these CAD tools can be safely and effectively used during childhood TB screening initiatives.

A key strength of this evaluation is the test library’s design and use of a composite reference standard. Most published CAD software evaluations have used a bacteriological reference standard, meaning they only evaluate software performance among the subset of participants who were tested during initial TB screening [9, 11, 12, 37, 38]. This can result in a bias and an underestimation of the CAD software’s accuracy and performance, as individuals with low-grade abnormalities and normal CXR results are often not indicated for diagnostic testing during TB screening. By including individuals with two normal CXR results in the test library, we have ensured that the test library reflects the demographics of the people who were initially screened in the community, so that it can better assess how a CAD software may perform under programmatic conditions. This is demonstrated by the Genki software achieving over 89% specificity when calibrated to 90% sensitivity using DICOM files—a level of performance that far exceeds that of any CAD software performance reported (Table A3.12) in the WHO’s revised CAD guidelines [16]. We acknowledge, however, that this community-based sample differs from the populations typically encountered in health facilities, where individuals often present with more advanced disease, and thus our findings may not fully capture CAD performance in such settings.

However, this evaluation and test library are not without limitations. Participant characteristics showed some unusual patterns, including a significantly lower rate of TB among individuals with HIV co-infection. This reflects the use of any HIV diagnosis as a basis for mobilization at the screening events which used the ultraportable radiography system, irrespective of viral load suppression status or CD4 count. The differences in target population mobilization between the ultraportable and truck-based mobile screening events, shaped by donor-defined objectives, contributed to the observed variation in TB rates. In addition, a normal CXR result does not preclude a TB diagnosis [39]. The use of two CXR interpretations from independent methods gives a higher confidence that individuals with two normal CXR results do not have TB. Yet, at the mobile CXR screening events which were the source of this evaluation’s test library data, a qXR software threshold of ≥ 0.5 (default system setting) was used to indicate additional Xpert-Ultra testing missed by the radiologist. A lower threshold of ≥ 0.2 or ≥ 0.3 would have indicated more people for testing, allowing for a higher proportion of the test library’s composite reference standard to be based on Xpert-Ultra testing, rather than radiological results. A single Xpert-Ultra test may also return a false negative result, particularly during community-based TB screening, where a large proportion of people with TB will likely have paucibacillary disease [40]. In addition, Xpert-Ultra testing after a history of TB can result in a false-positive result [41]. Together, these limitations suggest that some test library participants may have been inaccurately classified as not having TB. Future CAD evaluations should consider using a lower abnormality score cut-off threshold for systematically indicating additional Xpert-Ultra testing in parallel to the radiologist, implementing enhanced diagnostic approaches (e.g., double Xpert-Ultra and/or culture testing) and collecting data about time since past TB treatment to more accurately define the reference standard. Further analyses to explore for variations in Genki CAD software performance across key subgroups (e.g., young vs. old, male vs. female, HIV negative vs. positive) would be of great interest to the broader TB community. However, this evaluation’s test library includes only 58 individuals with TB and is therefore insufficiently powered to support reliable subgroup analyses, particularly when some key subgroups comprise fewer than 10 people with TB. Future evaluations with larger test libraries, or prospective monitoring during real-world deployments, will be essential to better understand subgroup-specific performance. As this evaluation was conducted independently of DeepTek, the specifics of JPEG image processing performed by the Genki CAD software remain unclear, including its potential impact on software performance. Nevertheless, the limited understanding of image processing algorithms does not compromise the validity of the evaluation’s findings.

## Conclusions

This evaluation demonstrates that Genki CAD software has high accuracy for TB screening when using either DICOM files or photographs of printed X-ray films, with performance surpassing that of human readers from the setting. The ability to process JPEG files, albeit under highly controlled conditions in this evaluation, may have the potential to broaden the software’s applicability, particularly in settings where digital infrastructure is limited. These findings support WHO’s recent endorsement of Genki as a recommended CAD software and highlight its potential to enhance TB screening coverage and quality. However, further prospective studies are needed to evaluate the feasibility and costs of its deployment and real-world effectiveness, as well as its performance during innovative applications and use cases. Continued evaluation across diverse populations, including children, and under varied operational conditions will be critical to fully realizing the value of this CAD software in accelerating global TB elimination efforts.

## Supplementary Information

Supplementary Material 1: Figure S1. Process flow for establishing a test library participant’s TB status. Table S1. Details about the human readers participating in the evaluation.

## Data Availability

The dataset used for this CAD software evaluation is publicly accessible at 10.5061/dryad.280gb5n3f [42].

## Abbreviations

AUC: Area under the curve
CAD: Computer-aided detection
CR: Computed radiography
CXR: Chest X-ray
DICOM: Digital Imaging and Communications in Medicine
DR: Digital radiography
JPEG: Joint Photographic Experts Group
ROC: Receiver operating characteristic
TB: Tuberculosis
TPP: Target product profile
WHO: World Health Organization
Xpert-Ultra: Xpert MTB/RIF Ultra assay
