# Supplementary material for: Bridging the analog divide: a comparison of printed X-ray films and digital images when using computer-aided detection software for tuberculosis screening
Source: BMC Glob Public Health. 2026 Jan 13;4:6. doi: 10.1186/s44263-025-00237-8 (PMC12801687; doi:10.1186/s44263-025-00237-8)
Supplement: Supplementary file 1 — Supplementary Material 1: Figure S1. Process flow for establishing a test library participant’s TB status. Table S1. Details about the human readers participating in the evaluation. [file 44263_2025_237_MOESM1_ESM.docx]

**Supplementary Material 1**


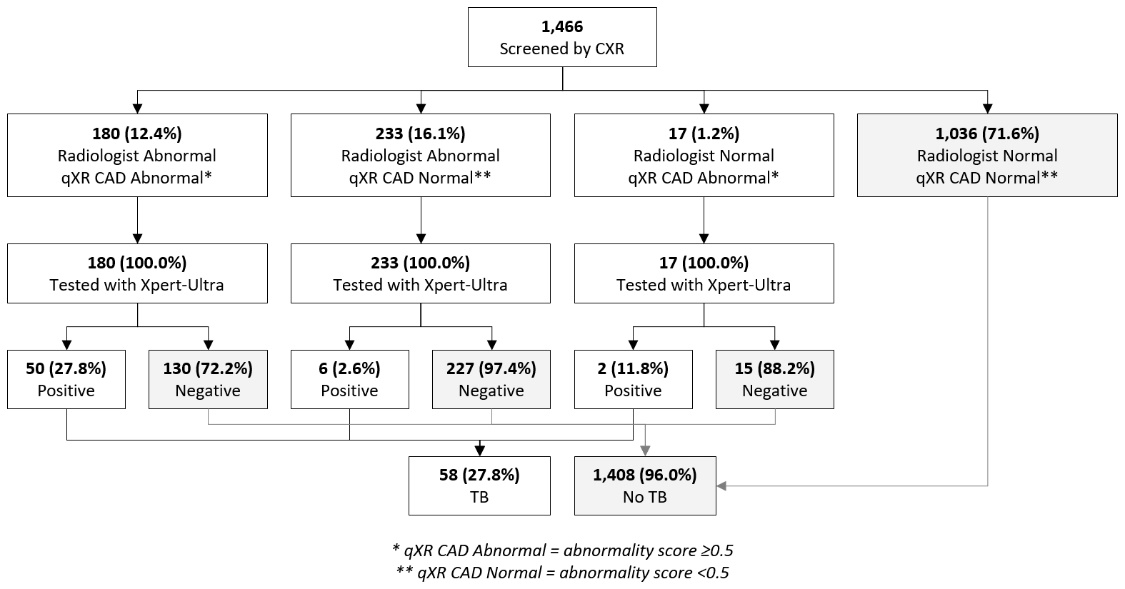


**Fig. S1**. Process flow for establishing a test library participant’s TB status

**Figure legend**: The 1,466 DICOM file test library participants are stratified into screening cohorts based on radiologist and qXR CAD software results, and follow-on Xpert-Ultra testing outcomes are shown. Final TB and No TB classifications are displayed at the bottom of the diagram.

**Table S1**. Details about the human readers participating in the evaluation

| **No.** | **Facility type** | **Medical specialization** | **Number of years working in respiratory/TB care** | **Radiology certificate (year last obtained)** |
| --- | --- | --- | --- | --- |
| 1 | Public | Family medicine | 10 | Yes (2013) |
| 2 | Public | TB | <1 | No |
| 3 | Public | TB | 13 | Yes (2018) |
| 4 | Private | Radiology | 34 | Yes (2009) |
| 5 | Private | Infectious diseases | 25 | No |
| 6 | Private | Pulmonology | 25 | Yes (2013) |
| 7 | Private | General practitioner | 3 | No |
| 8 | Private | Endocrinology | 6 | No |
| 9 | Public | Family medicine | 1 | Yes (2023) |
| 10 | Public | TB | 12 | No |
